# MicroRNA-455-3p promotes TGF-β signaling and inhibits osteoarthritis development by directly targeting PAK2

**DOI:** 10.1038/s12276-019-0322-3

**Published:** 2019-10-04

**Authors:** Shu Hu, Xiaoyi Zhao, Guping Mao, Ziji Zhang, Xingzhao Wen, Chengyun Zhang, Weiming Liao, Zhiqi Zhang

**Affiliations:** Department of Joint Surgery, the First Affiliated Hospital of Sun Yat-sen University, Guangzhou, 510080 China

**Keywords:** miRNAs, Osteoarthritis

## Abstract

MicroRNAs (miRNAs, miR) play a key role in the pathogenesis of osteoarthritis (OA). Few studies have examined the regulatory role of P21-activated kinases (PAKs), a family of serine/threonine kinases, in OA. The aim of this study was to determine whether miR-455-3p can regulate cartilage degeneration in OA by targeting PAK2. MiR-455-3p knockout mice showed significant degeneration of the knee cartilage. MiR-455-3p expression increased and PAK2 expression decreased in the late stage of human adipose-derived stem cell (hADSC) chondrogenesis and in chondrocytes affected by OA. Furthermore, in both miR-455-3p-overexpressing chondrocytes and PAK2-suppressing chondrocytes, cartilage-specific genes were upregulated, and hypertrophy-related genes were downregulated. A luciferase reporter assay confirmed that miR-455-3p regulates PAK2 expression by directly targeting the 3′-untranslated regions (3′UTRs) of PAK2 mRNA. IPA-3, a PAK inhibitor, inhibited cartilage degeneration due to OA. Moreover, suppressing PAK2 promoted R-Smad activation in the TGF/Smad signaling pathway in chondrocytes. Altogether, our results suggest that miR-455-3p promotes TGF-β/Smad signaling in chondrocytes and inhibits cartilage degeneration by directly suppressing PAK2. These results thus indicate that miR-455-3p and PAK2 are novel potential therapeutic agents and targets, respectively, for the treatment of OA.

## Arthritis: A molecular mechanism for cartilage maintenance

Functional insights into a short RNA strand that prevents cartilage degeneration could lead to new therapeutic strategies for treating osteoarthritis. The microRNA miR-455-3p regulates genes in tissues throughout the body, but Weiming Liao and Zhiqi Zhang of the First Affiliated Hospital of Sun Yat-sen University in Guangzhou, China have observed that it appears to play a particularly prominent role in cartilage-producing chondrocyte cells. Liao, Zhang and colleagues have now explored this mechanism in detail, and determined that miR-455-3p selectively blocks the effects of a protein called PAK2 in chondrocytes. PAK2 normally inhibits an important signaling pathway underlying cartilage generation, and such inhibition has previously been observed in MDCK epithelial cells. The authors conclude that this microRNA or other drugs that replicate its PAK2-inhibiting effects could prevent or slow the joint damage associated with this degenerative disorder.

## Introduction

Osteoarthritis (OA), characterized by osteophyte formation and articular cartilage degradation, is the leading cause of chronic disability in older adults^1^. However, the underlying pathogenesis is still unknown^2^. In recent years, microRNAs (miRNAs, miR) have attracted considerable attention due to their critical role in the regulation of gene expression through binding to the 3′-untranslated regions (3′UTRs) of target genes^3^. For example, miR-21 regulates the development of OA by targeting GDF-5^4^, whereas miR-139 induces apoptosis in OA chondrocytes by targeting MCPIP1^5^. We previously reported the upregulation of miR-455-3p during early chondrogenesis of human adipose-derived stem cells (hADSCs)^6^ and ATDC5 cells^7,8^. Subsequently, we hypothesized that miR-455-3p may play a key role in chondrogenesis or cartilage degeneration.

The TGF-β/Smad signaling pathway is an important regulator of cartilage anabolism. This pathway can enhance the synthesis of type II collagen and aggrecan, and prevent cartilage degradation^9,10^. TGF-β/Smad signaling is initiated by ligand binding to the type II receptor, which subsequently phosphorylates the type I receptors, both of which are serine/threonine kinase receptors. For canonical pathways, the activated receptor complex phosphorylates receptor-regulated Smad proteins (R-Smad: Smad2/3). The phosphorylated R-Smad then associates with Smad4 to form a complex that translocates into the nucleus, where it interacts with transcription factors to activate distinct subsets of genes.

P21-activated kinases (PAKs) are a family of serine/threonine kinases that are classified into two groups: groups I (PAK1-3) and II (PAK4-6). PAKs contain an autoinhibitory domain, which can be activated by the small GTP-binding proteins Cdc42 and Rac1^11,12^. PAKs have multiple biological functions^13^, and PAK2 is widely distributed throughout the body. Several substrates of PAK2, such as c-Jun, and cellular events associated with PAK2, including tumorigenesis, have been identified^14,15^. PAK2 also helps mediate COX2 expression in papillomas through the NF-κB pathway activation^16^. Moreover, PAK2 inhibits the TGF-β signaling pathway by interfering with the R-Smad interaction in Madin–Darby canine kidney (MDCK) epithelial cells^17^. However, no studies have assessed the effects of PAK2 on OA chondrocytes. Using miRNA target-prediction algorithms, we found that miR-455-3p potentially regulates PAK2 expression. Based on this information and the regulatory role of PAK2 in the TGF-β signaling pathway, we hypothesized that miR-455-3p may regulate the metabolism of chondrocytes by modulating PAK2 expression.

In this study, we report the role of PAK2 in OA chondrocytes and demonstrate that miR-455-3p promotes TGF-β signaling and inhibits cartilage degeneration in OA chondrocytes by directly targeting PAK2.

## Materials and methods

This study adhered to the standards of the Ethics Committee on Human Experimentation of The First Affiliated Hospital at Sun Yat-Sen University, China (IRB:2011011) and the Helsinki Declaration (2000). All participants provided informed consent.

### MiR-455-3p global knockout (KO) mouse model

All mouse breeding and animal procedures were approved by the Animal Research Committee of The First Affiliated Hospital of Sun Yat-sen University, China (IRB: 2014C-028). MiR-455-3p global KO mice were generated using a transcription activator-like effector nuclease (TALEN) system, which was described in our previous study^18^. Three pairs of KO and wild-type (WT) C57BL/6 mice were euthanized at either 5 months or 12 months of age, and their knee joints were collected. The knee joint sections were stained via immunohistochemistry and in situ hybridization for further analysis. After staining with Safranin O and Fast Green, the tissues were scored for histopathology by the modified Mankin scoring system^19^. The OA histopathology was quantified by summing the scores for the following four criteria: surface fissuring (0–3), pericellular matrix staining (0–2), spatial arrangement of chondrocytes (0–3), and interterritorial matrix staining (0–3). A higher score indicates more advanced OA histopathology.

### Cell isolation and culture

OA cartilage samples were obtained from the knee joint of patients with OA who underwent total knee replacement surgery (*n* = 6, mean ± standard deviation [SD] age: 62.83 ± 1.17 years, male: 1, female: 5), and control cartilage samples were obtained from patients with no history of OA or rheumatoid arthritis who underwent low limb amputation surgery due to sarcomas not involving the knee or ankle joints (*n* = 7, mean ± SD age: 19.29 ± 5.94 years, male: 4, female: 3). The isolation method of primary human chondrocytes (PHCs) from cartilage was described previously^20^. PHCs were cultured in Dulbecco’s modified Eagle’s medium F-12 (DMEM/F-12; Gibco Life Technology) supplemented with 5% fetal bovine serum (FBS; Gibco Life Technology) and 1% penicillin/streptomycin (Gibco Life Technology). The isolation method of hADSCs was described in our previous study^21^. Three adipose tissue samples were obtained from patients (mean age: 21 years, range: 16–30 years) who underwent abdominal surgery or elective liposuction. The hADSCs were cultured in alpha minimum essential medium (α-MEM; Gibco Life Technology) supplemented with 10% FBS. All cells were cultured in a humidified 5% CO_2_ atmosphere at 37 °C. When the cultures reached ~80% confluence, the cells were detached by treatment with 0.05% trypsin/ethylenediaminetetraacetic acid (EDTA) and passaged, and the culture media were changed every 3 days.

### Induction of chondrogenesis in hADSCs

The chondrogenesis induction method was described in our previous study^6^. In brief, cultured hADSCs were resuspended in incomplete chondrogenic medium (194 mL human adipose mesenchymal stem cell chondrogenic differentiation basal medium, 20 μL dexamethasone, 600 μL ascorbate, 2 mL of ITS [insulin, transferrin, and selenium] supplement, 200 μL sodium pyruvate, 200 μL proline; Cyagen, Guangzhou, China), seeded in 24-well plates at a density of 10^5^ cells/μL and incubated at 37 °C for 70 min. After incubation, 500 μl of complete chondrogenic medium (incomplete chondrogenic medium with 5 μL TGF-β3) was added to each well, and the complete medium was changed every 3 days. Samples were collected at different time points for the experiments.

### RNA extraction, reverse transcription, and qRT-PCR

The total RNA extraction was performed using the miRNeasy Mini Kit (QIAGEN, CA, USA), and cDNA was synthesized using the PrimeScript® miRNA cDNA Synthesis Kit (TaKaRa Bio, Japan). Quantitative real-time polymerase chain reaction (qRT-PCR) was performed using SYBR® Premix Ex Taq™ II (TaKaRa Bio, Japan) and a CFX96 real-time qPCR machine. The primers used for analysis are listed in Table 1. Relative gene expression was calculated using the 2^−ΔΔt^ method. All experiments were performed in triplicate.

**Table 1:** Primers for quantitative real-time polymerase chain reaction (qRT-PCR)

Gene		Primer sequence (5′-3′)
*has-GAPDH*	F	GCACCGTCAAGGCTGAGAAC
has-GAPDH	R	ATGGTGGTGAAGACGCCAGT
*has-SOX9*	F	AGCGAACGCACATCAAGAC
*has-SOX9*	R	CTGTAGGCGATCTGTTGGGG
has-COL2A1	F	TGGACGATCAGGCGAAACC
*has-COL2A1*	R	GCTGCGGATGCTCTCAATCT
*has-ACAN*	F	GTGCCTATCAGGACAAGGTCT
*has-ACAN*	R	GATGCCTTTCACCACGACTTC
*has-RUNX2*	F	CACTGGCGCTGCAACAAGA
*has-RUNX2*	R	CATTCCGGAGCTCAGCAGAATAA
*has-COL10A1*	F	CATAAAAGGCCCACTACCCAAC
*has-COL10A1*	R	ACCTTGCTCTCCTCTTACTGC
*has-MMP13*	F	TCCTGATGTGGGTGAATACAATG
*has-MMP13*	R	GCCATCGTGAAGTCTGGTAAAAT
*has-PAK2*	F	CAGAAACAGCCAAAGAAGGAAC
*has-PAK2*	R	AACGATGTTGGGATTTTTCAA
*has-U6*	F	CTCGCTTCGGCAGCACA
*has-U6*	R	AACGCTTCACGAATTTGCGT
*has-455-3P*	F	GCAGTCCATGGGCATATACAC

### Transfection

PHCs were transfected with a miR-455-3p mimic (RiboBio, Guangzhou, China) at a concentration of 50 nM or an inhibitor of miR-455-3p at a concentration of 100 nM. A nonspecific miRNA (miR-Control; RiboBio) was used as a control. PHCs were also transfected with siPAK2 (50 nM) and siNC (RiboBio) as negative controls. Lipofectamine® 2000 Transfection Reagent (Gibco Life Technologies) was used to transfect PHCs according to the manufacturer’s instructions.

### Western blot analysis

The protein collection and western blot protocols were described in our previous study^8^. The nuclear proteins were isolated using a Nuclear Extraction kit (CW0199, CoWin Biosciences). Briefly, 20 μg of protein was separated by sodium dodecyl sulfate-polyacrylamide gel electrophoresis (SDS-PAGE) and transferred to the polyvinylidene difluoride (PVDF) membranes (Millipore, Bedford USA). The membranes were incubated overnight at 4 °C with primary antibodies against PAK2 (1:1000 dilution, Cell Signaling Technology, #2615), phospho-PAK2 (1:100 dilution, Abcam, ab40795), RUNX2 (1:1000 dilution, Abcam, ab76956), GAPDH (1:1000 dilution, CST), PCNA (1:1000 dilution, Proteintech, 10205-2-AP), COL2A1 (1:1000 dilution, Abcam, ab188570), MMP13 (1:1000 dilution, Abcam, ab39012), SOX9 (1:2000 dilution, Millipore, ABE2868), Smad2 (1:1000 dilution, CST, #5339 S), Smad3 (1:1000 dilution, CST, #9523), phospho-Smad2 (1:1000 dilution, CST, #18338) and phospho-Smad3 (1:1000 dilution, CST, #9520). After incubation with primary antibodies, the membranes were incubated with the corresponding horseradish peroxidase (HRP)-conjugated secondary antibodies (1:3000 dilution, Cell Signaling Technology) at room temperature for 1 h.

### Immunohistochemical analysis and in situ hybridization

The methods for immunohistochemistry and in situ hybridization were described in our previous study^20,22^. For in situ hybridization, a probe for human miR-455-3p (Exiqon, Invitrogen, Shanghai, China) was used. For immunohistochemical analysis, deparaffinization and rehydration of the sections were performed with standard xylene-to-ethanol washes. The sections were then blocked in phosphate-buffered saline (PBS) plus 0.025% Tween 20 with 10% FBS. After blocking, the sections were incubated at 4 °C overnight with primary antibodies specific for PAK2 (1:100 dilution, Abcam, ab76293), phospho-PAK2 (1:100 dilution, Abcam, ab40795), COL2A1 (1:100 dilution, Abcam, ab188570), and MMP13 (1:80 dilution, Abcam, ab39012). Negative controls were prepared by substituting PBS for the primary antibodies. After overnight incubation, the sections were incubated with HRP-conjugated anti-rabbit IgG secondary antibody (Cell Signaling Technology, Boston, USA) for 30 min.

### Cell viability assay

OA chondrocytes (2000 cells/well) were seeded into 96-well plates. When the cultures reached ~80% confluence, the supernatant was removed, and media with different concentrations of IPA-3 were added to the OA chondrocytes. At the indicated time points, the cell viability was measured using a Cell Counting Kit-8 (CCK-8, Dojindo Laboratories, Kumamoto, Japan) according to the manufacturer’s instructions.

### Luciferase constructs and luciferase reporter assay

PAK2 3′UTR DNA was amplified by PCR using the forward primer 5′-ATAGGCCGGCATAGACGCGTCATCACTGCTGTGGCCTCATACTC-3′ and the reverse primer 5′-AAAGATCCTTTATTAAGCTTGGGGAGGGGAGAAGGGAGG-3′. Seed sequences were mutated using PCR with the forward primer 5′-TAGTGATCTTTCGTGCAATTCCTTCTGGACCCTAAAGAAGG-3′ and the reverse primer 5′-AGAAGGAATTGCACGAAAGATCACTAGCCTTAGGTCTTTCAGCAAAC-3′. The amplified DNA sequences were inserted into the pmiR-RB-REPORT™ Vector (OBIO, Shanghai, China) to generate WT or mutant PAK2 3′UTR luciferase vectors. For the luciferase reporter assay, 1.2 × 10^4^ cells (HEK293) were cotransfected with 100 nM miR-455-3p or miR-Control and 0.2 μg of vector containing WT or mutant PAK2 3′UTR in a 96-well plate. After 48 h of transfection, the Dual-Luciferase® Reporter Assay System (Promega Corp, Madison, WI, USA) was used to measure luciferase activity. Firefly luciferase activity was normalized to the Renilla luciferase activity. Luciferase assays were performed in quadruplicate and repeated in three independent experiments.

### Statistical analysis

All experiments were performed with at least three biological replicates, and the data are presented as the mean ± standard deviation (SD). Student’s *t* tests or Mann–Whitney U tests were used to identify differences between groups. The Gaussian distribution of the data was confirmed using the Shapiro–Wilk test. One-way analysis of variance (ANOVA) and Kruskal–Wallis tests were carried out for multiple group comparisons. *P*-values of <0.05 were considered statistically significant. All analyses were performed using SPSS software, version 13.0 (IBM Corporation, Armonk, NY, USA).

## Results

### Knee cartilage phenotype in miR-455-3p knockout mice

**Fig. 1:**
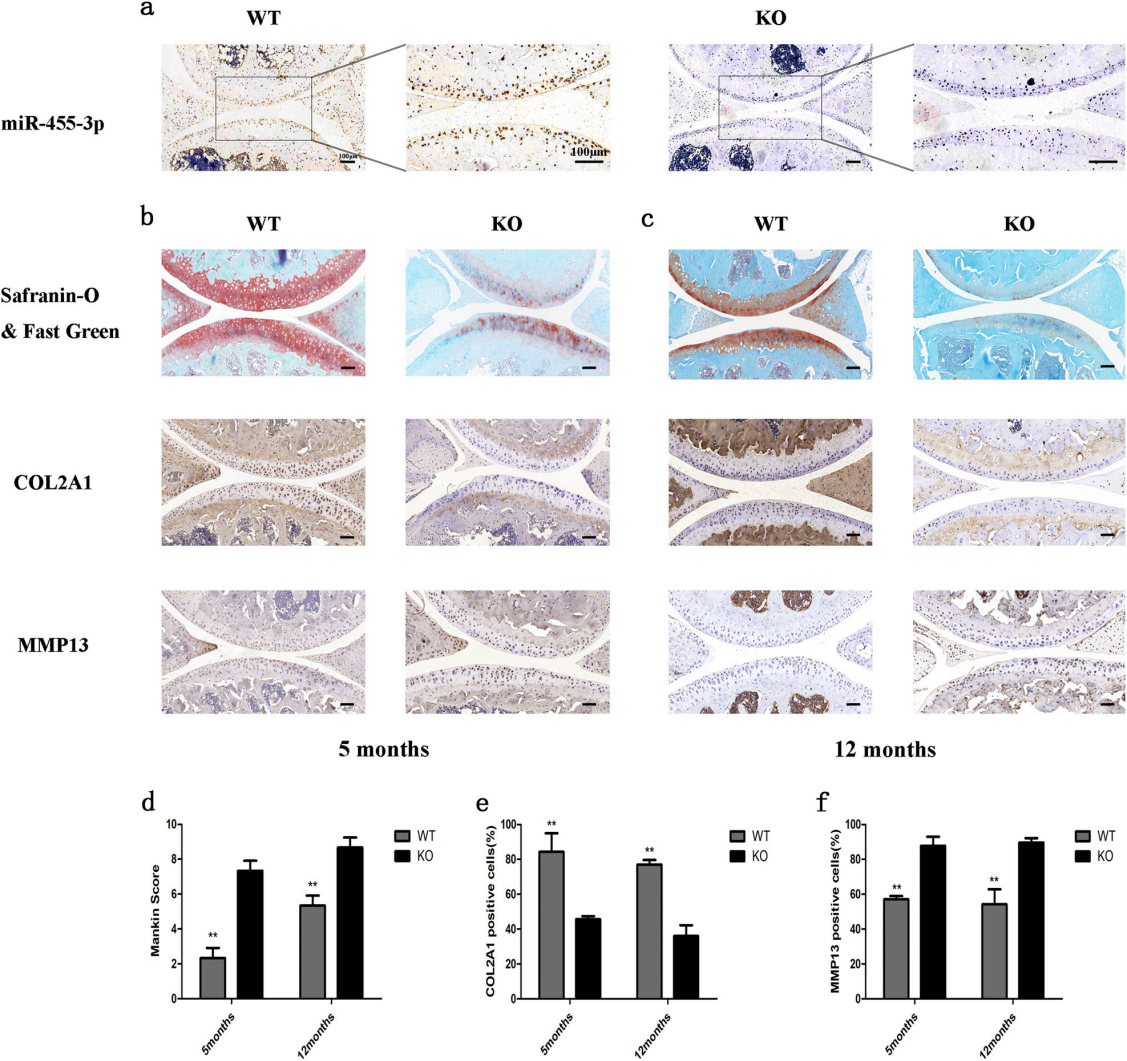
Phenotypes of miR-455-3p knockout mice at 5 months and 12 months of age. The knee joints of miR-455-3p KO mice and WT mice were collected at 5 months and 12 months of age. In situ hybridization targeting miR-455-3p was performed to verify the knockout efficiency in 12-month-old KO and WT mice (**a**). Safranin O and Fast Green staining and immunohistochemistry for COL2A1 and MMP13 were performed with samples from 5-month-old mice (**b**, **e**, **f**) and 12-month-old mice (**c**, **e**, **f**). KO and WT mice were scored for histopathology according to the modified Mankin scoring system (**d**). Magnified view at ×100 magnification; areas enclosed by black boxes are shown at ×200 magnification. The data shown are representative results from experiments conducted with three pairs of wild-type and miR-455-3p-deletion mice. Quantitative data are represented as the mean ± SD from three pairs of KO and WT mice. Scale bar: 100 μm. *p *<* 0.05, **p *<* 0.01

To investigate the role of miR-455-3p in cartilage development, we compared 5-month-old and 12-month-old miR-455-3p-deletion mice and wild-type mice. As shown in Fig. 1a, in situ hybridization of miR-455-3p confirmed the deletion efficiency in 12-month-old wild-type and mutant mice. MiR-455-3p was significantly decreased in KO mouse cartilage. In addition, the miR-455-3p KO mice showed surface irregularities on the articular surfaces of the knee joint, increased hypocellularity, and decreased Safranin O staining of the extracellular matrix compared with the WT mice. The Mankin score of the miR-455-3p mice was significantly higher than that of the WT mice at both 5 and 12 months of age (Fig. 1b–d). COL2A1 levels were significantly reduced in the miR-455-3p-deletion mice at 5 months and 12 months, while MMP13 expression was substantially increased in the mutant mice (Fig. 1b, c, e, f). These results indicated that miR-455-3p plays a crucial role in cartilage development and degeneration.

### Expression patterns of miR-455-3p and PAK2 during chondrogenesis of hADSCs

**Fig. 2:**
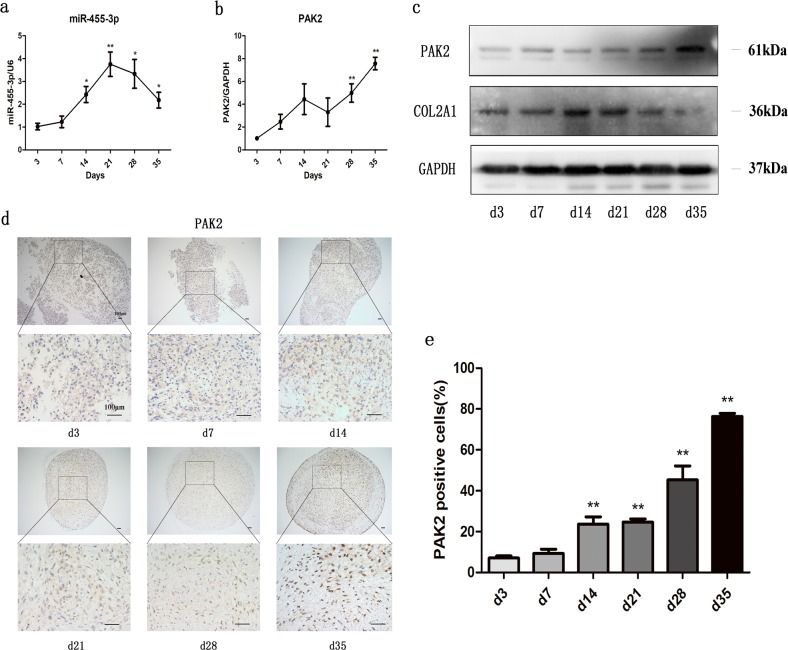
Relative expression levels of miR-455-3p and PAK2 during the chondrogenesis of hADSCs. TGF-β3-induced chondrogenesis of hADSCs. Gene expression of miR-455-3p (**a**) and PAK2 (**b**) was evaluated by qRT-PCR at days 3, 7, 14, 21, 28, and 35. Protein levels of PAK2 and COL2A1 were determined by western blotting (**c**). Chondrogenesis of hADSCs was evaluated by immunohistochemistry for PAK2 (**d**–**e**). The upper panels are at ×100 magnification, while the lower panels show ×400 views of the areas enclosed by black boxes. The data shown represent three independent experiments with samples from three different donors. The quantitative data are represented as the mean ± SD. Scale bar: 100 μm. *p < 0.05, **p < 0.01

To investigate the regulatory mechanism of miR-455-3p in chondrocytes and confirm our hypothesis, we studied this mechanism in vitro. Chondrogenesis of hADSCs was induced by TGF-β3. As shown in Fig. 2a, miR-455-3p expression increased rapidly at the beginning of day 3, peaked at day 21, and then sharply decreased from day 28 to day 35. However, an opposite expression pattern was observed for PAK2 and miR-455-3p during chondrogenic differentiation from days 14 to 35 (Fig. 2b–e), indicating that the expression levels of miR-455-3p and PAK2 may be related to cartilage degeneration and that the expression of PAK2 may be affected by miR-455-3p. Finally, chondrogenesis of hADSCs was confirmed by the expression levels of COL2A1, which increased during early chondrogenesis and decreased in late-stage chondrogenesis (Fig. 2c).

### Expression levels of miR-455-3p and PAK2 in human OA and control cartilage

**Fig. 3:**
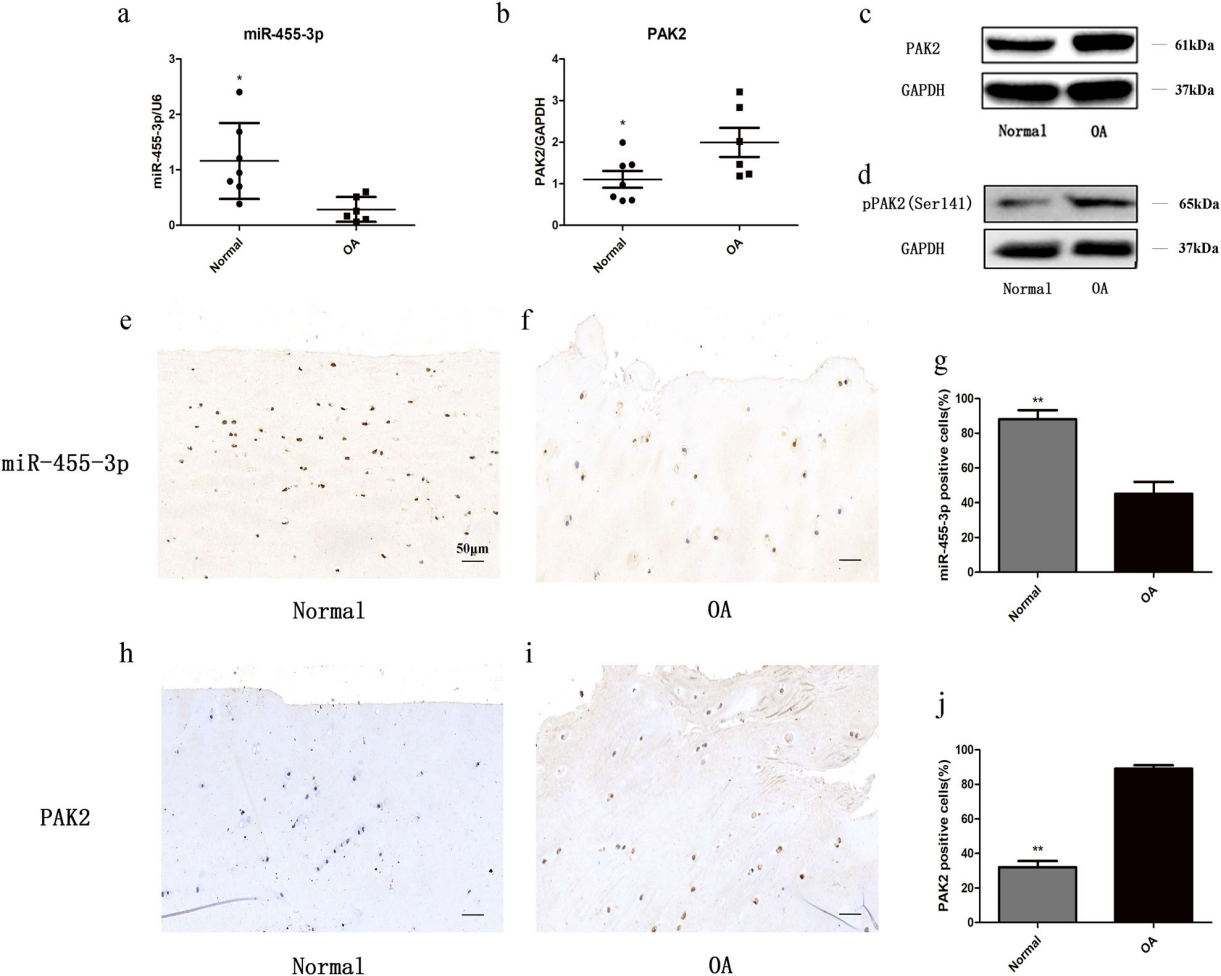
Expression levels of miR-455-3p and PAK2 in OA and control chondrocytes. The expression levels of miR-455-3p (**a**) and PAK2 (**b**) were determined by qRT-PCR. U6 and glyceraldehyde 3-phosphate dehydrogenase (GAPDH) were used as endogenous controls, and each dot represents a value from a single experiment with one donor sample. The miR-455-3p levels in OA and control cartilage were determined by in situ hybridization (**e**–**g**). The protein levels of phospho-PAK2 were determined by western blotting (**d**). The protein levels of PAK2 were determined by western blotting (**c**) and immunohistochemistry (**h**–**j**). GAPDH was used as an endogenous control. The data shown are representative results from six control and OA cartilage samples, and images are shown at ×200 magnification. Scale bar: 50 μm. *p *<* 0.05, **p *<* 0.01

To further determine whether the expression levels of miR-455-3p and PAK2 changed during cartilage degeneration, we compared the expression levels of miR-455-3p and PAK2 in human control and OA cartilage by qRT-PCR and western blotting. The miR-455-3p levels were decreased, and both PAK2 and phospho-PAK2 were increased in OA cartilage compared with control cartilage (Fig. 3a–d). This trend was confirmed by in situ hybridization (Fig. 3e–g) and immunohistochemical analysis (Fig. 3h–j). These results further indicated that miR-455-3p and PAK2 are involved in OA progression and that PAK2 may be regulated by miR-455-3p.

### PAK2 knockdown has effects similar to those of miR-455-3p overexpression on OA chondrocytes

**Fig. 4:**
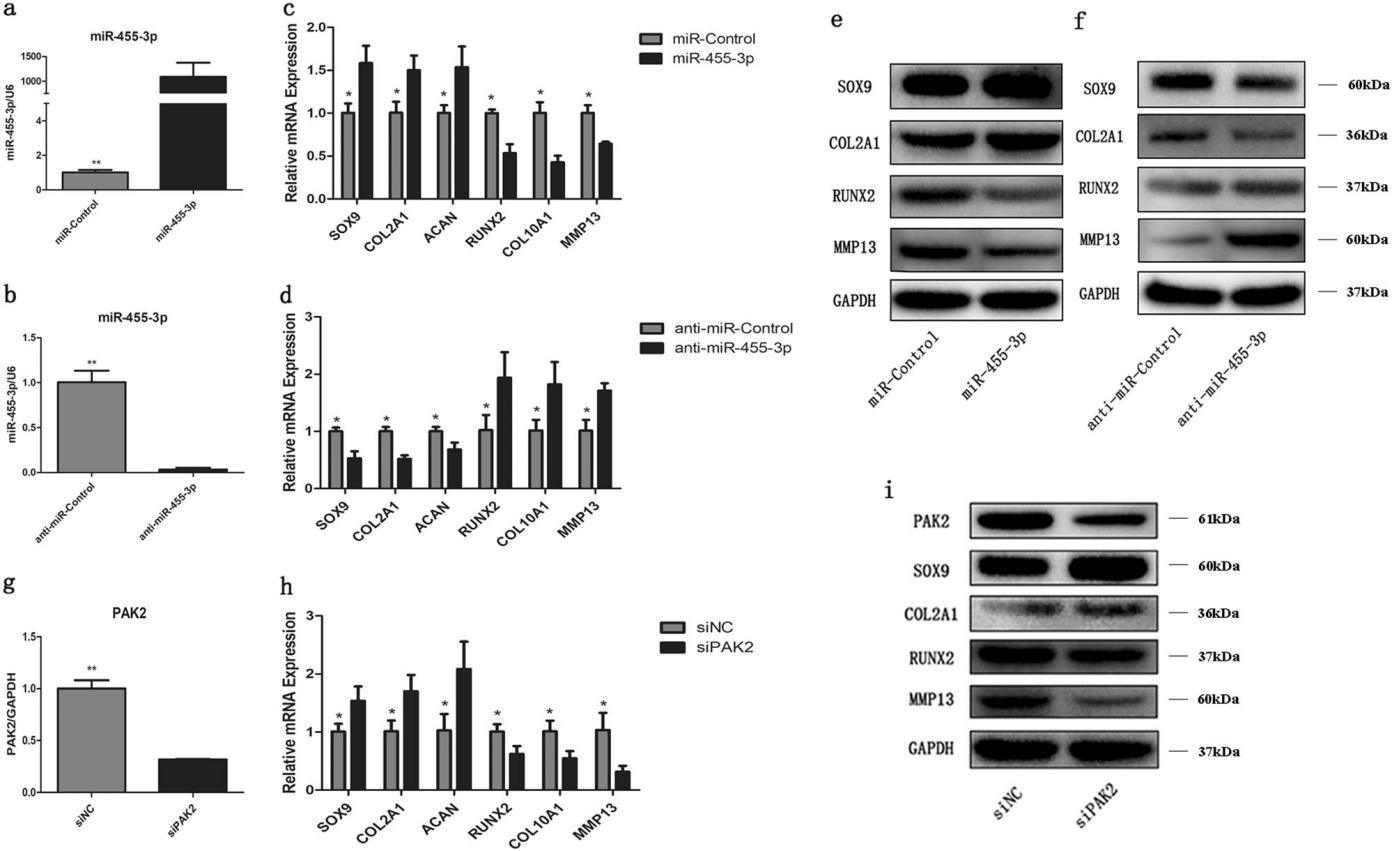
MiR-455-3p and PAK2 regulate the expression of cartilage-specific genes in OA chondrocytes. After 48 h of transfection with miR-455-3p, miR-Control, anti-miR-455-3p, anti-miR-Control, and/or PAK2 siRNA, the transcript levels of miR-455-3p, PAK2, SOX9, COL2A1, ACAN, RUNX2, COL10A1, and MMP13 were measured by qRT-PCR (**a**–**d**, **g**, **h**). GAPDH and U6 were used as endogenous controls. After 72 h of transfection, the protein levels of PAK2, SOX9, COL2A1, RUNX2, and MMP13 were analyzed by western blotting (**e**, **f**, **i**). GAPDH was used as an internal control. Quantitative data are represented as the mean ± SD from three independent experiments. *p < 0.05, **p *<* 0.01

To further investigate the role of miR-455-3p and PAK2 in OA cartilage, we transfected OA chondrocytes with miR-455-3p, anti-miR-455-3p, and/or siPAK2. After transfection, the expression levels of cartilage-specific genes (SOX9, COL2A1, and ACAN) and hypertrophy-related genes (RUNX2, COL10A1, and MMP13) were determined by qRT-PCR, and the protein levels of SOX9, COL2A1, RUNX2, and MMP13 were assessed by western blotting. Overexpression of miR-455-3p in OA chondrocytes promoted the expression of cartilage-specific genes and decreased the expression of hypertrophy-related genes. In contrast, an opposing expression pattern of these genes was observed in OA chondrocytes with miR-455-3p silencing (Fig. 4a–d). Similar relationships between miR-455-3p and the expression levels of SOX9, COL2A1, RUNX2, and MMP13 were detected by western blotting (Fig. 4e–f). The knockdown of PAK2 was correlated with increased expression of cartilage-specific genes, whereas the expression of RUNX2, COL10A1, and MMP13 was downregulated (Fig. 4g–i). These results indicated that the level of miR-455-3p or PAK2 can regulate cartilage degeneration.

### MiR-455-3p suppresses PAK2 expression by directly targeting the PAK2 3′UTR

**Fig. 5:**
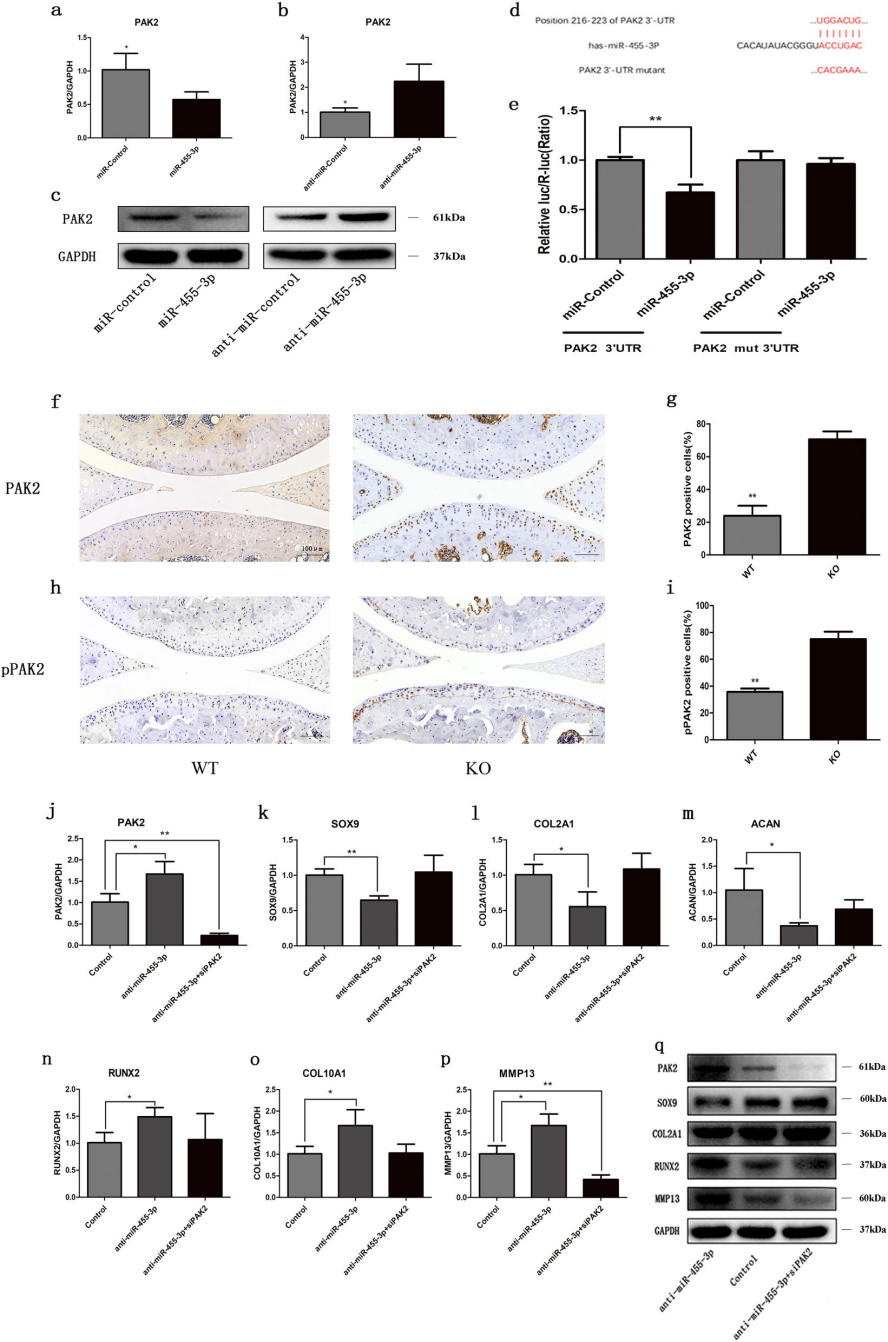
MiR-455-3p regulates the expression of cartilage-specific genes by targeting PAK2. OA chondrocytes were transfected with miR-455-3p, miR-Control, anti-miR-455-3p, anti-miR-Control, or anti-miR-455-3p + siPAK2. The transcript levels of PAK2, SOX9, COL2A1, ACAN, RUNX2, COL10A1, and MMP13 were measured by qRT-PCR after 48 h of transfection (**a**, **b**, **j**–**p**). The protein levels of PAK2, SOX9, COL2A1, RUNX2, and MMP13 were visualized by western blotting after 72 h of transfection (**c**, **q**). The sequence alignment of miR-455-3p and PAK2 3′UTR is shown (**d**). First, 293T cells were cotransfected with either a WT or mutant PAK2 3′UTR reporter plasmid and either a miR-455-3p or anti-miR-455-3p construct. Cells were harvested for luciferase assays after 48 h of transfection (**e**). Immunohistochemistry analysis of PAK2 (**f**–**g**) and phospho-PAK2 (**h**–**i**) was performed in 12-month-old wild-type and miR-455-3p knockout mice. Magnified view at ×150 magnification. Quantitative data are represented as the mean ± SD from three independent experiments. GAPDH was used as an internal control. Scale bar: 100 μm. *p *<* 0.05, **p *<* 0.01

Because of the similar effects of siPAK2 and the miR-455-3p mimic, we hypothesized that miR-455-3p may regulate the degeneration of cartilage through downregulation of PAK2. PAK2 mRNA and protein levels were decreased in OA chondrocytes overexpressing miR-455-3p and were increased upon addition of anti-miR-455-3p (Fig. 5a–c). To investigate the molecular mechanisms underlying the regulation of PAK2 expression by miR-455-3p, we analyzed the 3′UTR of human PAK2 mRNA. Web servers that predicts biological targets of miRNAs, such as TargetScan (http://www.targetscan.org) and miRanda (http://www.microrna.org), revealed that the 3′UTR of human PAK2 contains a potential binding site for miR-455-3p (Fig. 5d). We therefore utilized a luciferase reporter assay to test this potential interaction. Cotransfection of miR-455-3p and PAK2 3′UTR luciferase reporter plasmids significantly reduced the luciferase activity, whereas a mutated PAK2 3′UTR sequence prevented this reduction (Fig. 5e). We further confirmed this regulatory relationship between miR-455-3p and PAK2 in vivo. The expression of PAK2 (Fig. 5f, g) and phospho-PAK2 (Fig. 5h, i) was significantly increased in miR-455-3p KO mice compared with WT mice at 12 months of age. These results confirmed that miR-455-3p regulates PAK2 expression by binding the PAK2 3′UTR.

### PAK2 knockdown blocks the effects of the miR-455-3p inhibitor on OA chondrocytes

To confirm that miR-455-3p regulates chondrocyte metabolism by targeting PAK2, we cotransfected OA chondrocytes with siPAK2 and anti-miR-455-3p to examine whether the effects of the miR-455-3p inhibitor could be blocked by PAK2 knockdown. We found that the miR-455-3p inhibitor decreased the expression of cartilage-specific genes and increased the expression of hypertrophy-related genes and that PAK2 siRNA significantly blocked these effects (Fig. 5j–p). Similar results were observed with western blotting (Fig. 5q). These results indicated that miR-455-3p regulates OA chondrocytes through targeting PAK2.

### Effects of IPA-3, a PAK inhibitor, on OA chondrocytes

**Fig. 6:**
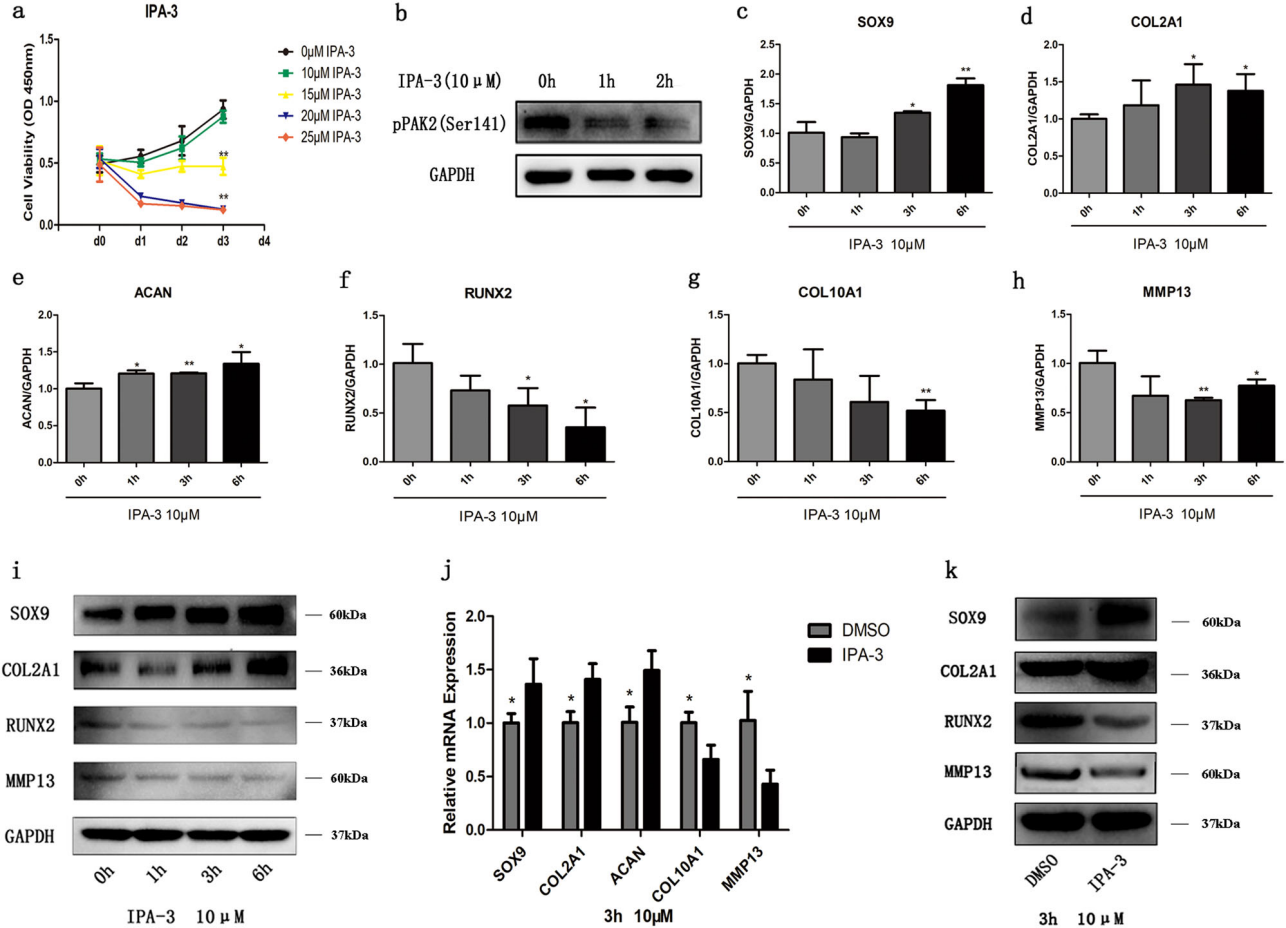
IPA-3 regulates the expression of cartilage-specific genes in OA chondrocytes. OA chondrocytes were incubated with different concentrations of IPA-3. Cell viability was detected with CCK-8 assays at d0 – d3 (**a**). OA chondrocytes were incubated with 10 μM IPA-3, and the level of phospho-PAK2 was detected by western blotting after 1 h and 2 h of treatment (**b**). The transcript levels of SOX9, COL2A1, ACAN, RUNX2, COL10A1, and MMP13 were measured by qRT-PCR at 0 h, 1 h, 3 h, and 6 h of IPA-3 treatment (**c**–**i**). The protein levels of SOX9, COL2A1, RUNX2, and MMP13 were visualized using western blotting (**i**). OA chondrocytes were incubated with 10 μM IPA-3 or DMSO, and the levels of transcripts and proteins were measured after 3 h of incubation (**j**–**k**). Quantitative data are represented as the mean ± SD from three independent experiments. GAPDH was used as an internal control. *p *<* 0.05, **p *<* 0.01

To further assess the role of PAK2 in cartilage, we treated OA chondrocytes with IPA-3, an allosteric PAK inhibitor^23^. Since 10 μM IPA-3 had no effect on the viability of OA chondrocytes after 3 days of treatment (Fig. 6a), this concentration was utilized for subsequent experiments. First, PAK2 phosphorylation levels were reduced by IPA-3 treatment (Fig. 6b). Furthermore, 10 μM IPA-3 increased the mRNA levels of SOX9, COL2A1, and ACAN, and decreased those of RUNX2, COL10A1, and MMP13 in a time-dependent manner (Fig. 6c–h). Similar trends were observed with western blotting (Fig. 6i). IPA-3 was dissolved in dimethyl sulfoxide (DMSO), and we used the same volume of DMSO for 3 h as a control; DMSO likely has no additional effects during the IPA-3 treatment (Fig. 6j, k). We further confirmed this finding through a time-course experiment in chondrocytes treated with DMSO (Supplementary Fig. 1). These results thus demonstrate that inhibiting PAK2 has positive effects on OA chondrocytes.

### IPA-3 and PAK2 knockdown promote TGF-β-induced R-Smad activation and signaling in chondrocytes

**Fig. 7:**
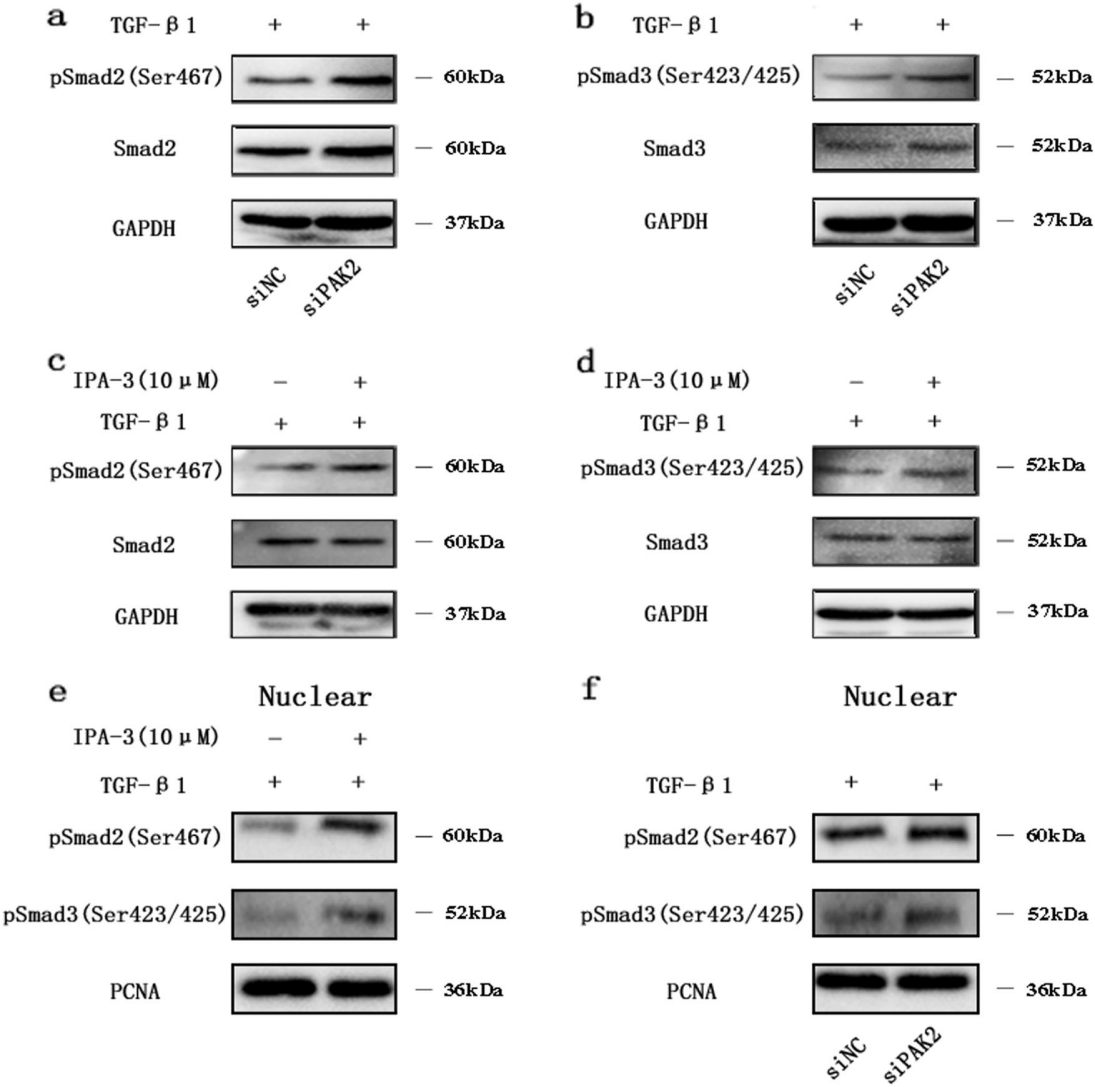
PAK2 inhibition promotes TGF-β signaling in chondrocytes. PHCs were transfected with siPAK2 or siControl for 48 h (**a**, **b**, **f**) and then treated with TGF-β1 for 1 h. After incubation with 10 μM IPA-3 for 3 h, PHCs were treated with 5 ng/ml TGF-β1 for 1 h (**c**, **d**, **e**). The total and nuclear levels of p-Smad2 and p-Smad3 proteins were analyzed by western blotting. Smad2 and Smad3 were used as internal controls. The data shown are representative results from three independent experiments

To investigate whether PAK2 can affect the TGF-β/Smad signaling pathway in chondrocytes, we transfected siPAK2 and siControl into PHCs. After 72 h of transfection, PAK2 knockdown increased the total and nuclear levels of phosphorylated Smad2 and Smad3 after 1 h of incubation with TGF-β1 (5 ng/ml) (Fig. 7a, b, f). A similar trend was found in the PHCs treated with IPA-3 (10 μM) compared with control cells (Fig. 7c, d, e). These results indicated that PAK2 is an antagonist of the TGF-β/Smad signaling pathway in chondrocytes.

## Discussion

MiR-455-3p has attracted considerable attention in recent years due to its regulatory functions in most tissues and organs of the human body^24,25^. In our previous study, we demonstrated that miR-455-3p regulates chondrogenesis in ATDC5^8^. However, the regulatory function of miR-455-3p in human OA chondrocytes is still unknown. In this study, we showed that miR-455-3p promotes TGF-β signaling and inhibits cartilage degeneration in human chondrocytes by directly targeting PAK2.

The TGF-β/Smad signaling pathway has been shown to play a key role in maintaining articular cartilage^26^. The levels of SOX9, COL2A1, and ACAN increased and those of RUNX2, COL10A1, and MMP13 decreased after activation of the TGF-β/Smad signaling pathway in chondrocytes^27–30^. Inhibiting the TGF-β/Smad signaling pathway in chondrocytes led to hypertrophic differentiation of chondrocytes and progressive development of OA^31,32^. Our results using miR-455-3p knockout mice showed that both miR-455-3p and the TGF-β/Smad signaling pathway are likely involved in cartilage development. As reported in a previous study on PAK2-mediated inhibition of the TGF-β signaling pathway^17^, PAK2 phosphorylates Smad2 at Ser417 and Smad3 at Ser375, thus abolishing TGF-β-induced Smad2/3 activation and downstream signaling. Based on the bioinformatics predictions of miR-455-3p and PAK2 interactions, we hypothesized that miR-455-3p could promote TGF-β/Smad signaling and inhibit cartilage degeneration by directly targeting PAK2.

We tested our hypothesis and found opposite expression patterns of miR-455-3p and PAK2 in the late stage of chondrogenesis of hADSCs. Contrasting expression trends of miR-455-3p and PAK2 were also observed in OA and control chondrocytes. These results indicated that decreased miR-455-3p levels and increased PAK2 levels may drive OA development. We then confirmed the effects of miR-455-3p overexpression or PAK2 knockdown on OA chondrocytes and observed increased SOX9, COL2A1, and ACAN expression, and decreased RUNX2, COL10A1, and MMP13 expression. These effects were similar to those observed upon activation of the TGF-β/Smad signaling pathway in chondrocytes. Moreover, these results further confirmed the importance of miR-455-3p and PAK2 in OA development. In addition, due to the opposite expression patterns and similar effects of these molecules, we proposed that miR-455-3p can regulate the expression of PAK2 by directly targeting its 3′UTR sequence. A luciferase reporter assay was performed to clarify the underlying regulatory mechanism. In addition, anti-miR-455-3p and siPAK2 were cotransfected into OA chondrocytes to confirm that the regulatory functions of miR-455-3p in OA chondrocytes are mediated by targeting PAK2. We also used IPA-3, a PAK inhibitor, to further clarify the role of PAK2 in OA chondrocytes. Finally, we determined the levels of p-Smad2 and p-Smad3 to demonstrate that inhibiting PAK2 can promote TGF-β/Smad signaling in chondrocytes. Based on our results, we concluded that miR-455-3p inhibits cartilage degeneration via suppressing PAK2 expression and promoting the activity of the TGF-β/Smad signaling pathway. A previous study^33^ demonstrated that miR-455-3p suppresses the Smad2/3 pathway, as shown by luciferase assays. However, these experiments were performed in SW-1353 chondrosarcoma cells, not in chondrocytes, which may be the reason for the discrepancy between the results of the two studies.

This study has several limitations. First, the reasons for miR-455-3p and PAK2 overexpression in the early stages of hADSC chondrogenesis were not clarified. Second, IPA-3 is not a PAK2-specific inhibitor. Although it would have been more appropriate to use a PAK2-specific inhibitor, IPA3 has also been widely used in previous PAK2 studies^17,34^. In addition, while our findings demonstrated that miR-455-3p may be an important factor in OA development through KO mice, future investigation of the destabilization of the medial meniscus model (DMM) in miR-455-3p KO mice is needed to further characterize the role of miR-455-3p in OA progression. This study also demonstrated that miR-455-3p regulates the TGF-β signaling pathway by targeting PAK2; however, further studies will be needed to provide more direct evidence that miR-455-3p is an upstream regulator of the TGF-β signaling pathway. Finally, to our knowledge, there has been little research conducted on PAK2 in OA. We performed primary research on the role of PAK2 in OA chondrocytes. The regulatory mechanism of PAK2 in OA requires further study.

Altogether, our results indicate that miR-455-3p can promote the TGF-β/Smad signaling pathway in chondrocytes and inhibit cartilage degeneration by directly suppressing PAK2. Thus, miR-455-3p may serve as a novel therapeutic agent and PAK2 a therapeutic target in OA.

## Supplementary information

Supplement Figure 1
