# Supplementary material for: MicroRNA-455-3p promotes TGF-β signaling and inhibits osteoarthritis development by directly targeting PAK2
Source: Exp Mol Med. 2019 Oct 4;51(10):118. doi: 10.1038/s12276-019-0322-3 (PMC6802609; doi:10.1038/s12276-019-0322-3)
Supplement: Supplementary file 1 — Supplement Figure 1 [file 12276_2019_322_MOESM1_ESM.docx]

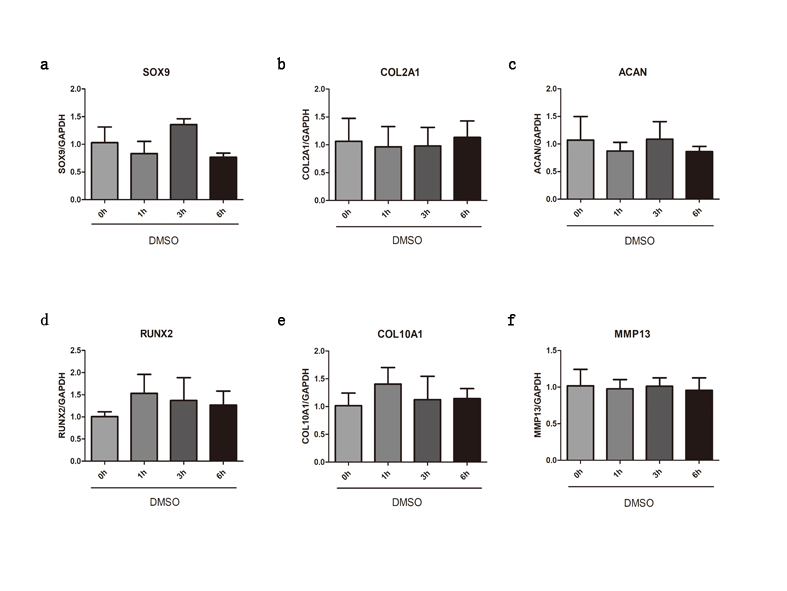


**Supplement Figure 1. Expression of cartilage-specific genes in OA chondrocytes after treatment with DMSO.** OA chondrocytes were treated with DMSO (same volume as 10 μM IPA-3) for 0 h, 1 h, 3 h, and 6 h. Transcript levels of SOX9, COL2A1, ACAN, RUNX2, COL10A1, and MMP13 were measured by qRT-PCR at each time point (a-f). Quantitative data are represented as the mean ± SD from three independent experiments. GAPDH was used as an internal control. * *p* < 0.05, ** *p* < 0.01.
